# *In vitro* performance of echoPIV for assessment of laminar flow profiles in a carotid artery stent

**DOI:** 10.1117/1.JMI.8.1.017001

**Published:** 2021-01-13

**Authors:** Astrid M. Hoving, Jason Voorneveld, Julia Mikhal, Johan G. Bosch, Erik Groot Jebbink, Cornelis H. Slump

**Affiliations:** a University of Twente, TechMed Centre, Robotics and Mechatronics Group, Enschede, The Netherlands; b Erasmus MC, Thorax Center, Department of Biomedical Engineering, Rotterdam, The Netherlands; c University of Twente, TechMed Centre, BIOS Lab-on-a-Chip Group, Enschede, The Netherlands; d University of Twente, TechMed Centre, Multi-Modality Medical Imaging Group, Enschede, The Netherlands

**Keywords:** high-frame-rate ultrasound, particle image velocimetry, carotid, stent, *in vitro*

## Abstract

**Purpose**: Detailed blood flow studies may contribute to improvements in carotid artery stenting. High-frame-rate contrast-enhanced ultrasound followed by particle image velocimetry (PIV), also called echoPIV, is a technique to study blood flow patterns in detail. The performance of echoPIV in presence of a stent has not yet been studied extensively. We compared the performance of echoPIV in stented and nonstented regions in an *in vitro* flow setup.

**Approach**: A carotid artery stent was deployed in a vessel-mimicking phantom. High-frame-rate contrast-enhanced ultrasound images were acquired with various settings. Signal intensities of the contrast agent, velocity values, and flow profiles were calculated.

**Results**: The results showed decreased signal intensities and correlation coefficients inside the stent, however, PIV analysis in the stent still resulted in plausible flow vectors.

**Conclusions**: Velocity values and laminar flow profiles can be measured *in vitro* in stented arteries using echoPIV.

## 1 Introduction

Carotid artery stenosis and rupture of atherosclerotic plaque result in a high risk of stroke or transient ischemic attack. Therefore, in case a patient suffers from severe carotid artery stenosis, treatment is indicated. The plaque can be removed surgically, or a stent can be placed to reopen the artery during a minimally invasive procedure. Currently, surgery is preferred over stenting, due to the higher peri-procedural stroke risk of stenting compared with surgery.^1^^,^^2^ However, long-term outcomes of stenting are comparable with those of surgery.^3^^,^^4^ This suggests that technical developments to the peri-procedural process might improve the stenting procedure. Investigation into carotid artery stenting is therefore of interest.

Analysis and improvement of carotid artery stenting can be supplemented by investigation of possible blood flow pattern changes after stent insertion. Disturbed blood flow results in decreased or oscillating wall shear stresses, leading to vessel wall remodeling. This increases the risk of atherosclerosis and stenosis.^5^ In case of stenting, the vessel wall remodeling caused by blood flow disturbances might result in in-stent stenosis. To capture the possible blood flow disturbances, detailed and quantitative information is required, provided by 2D or 3D vector fields obtained from data with high spatial and temporal resolution.

Investigation into imaging techniques to visualize detailed flow patterns in the presence of a stent is necessary, since current vascular imaging techniques have certain drawbacks. CT angiography does not provide velocity vector fields, and moreover it subjects the patient to harmful radiation and contrast agents. Magnetic resonance imaging suffers from strong metal artifacts in the area of the stent.^6^ Duplex ultrasound, the clinical standard to measure blood velocity by combining traditional and Doppler ultrasound, has an angle dependency, and flow information is limited to velocities in a specific sample volume inside the artery.^7^^,^^8^

To our knowledge, there are five ultrasound imaging techniques that are promising for studying flow patterns in a carotid artery stent. Vector Doppler, where two (or more) apertures are acquired to obtain both the axial and lateral velocity components, overcomes the limitation of Doppler ultrasound of measuring velocity in only one direction.^9^^–^^12^ Another technique applies transverse oscillations, i.e., perpendicular to the beam direction, to capture lateral components of the flow.^13^^–^^15^ An alternative, directional velocity estimation, is a technique where beam formation at multiple angles is used to find the angled frame with the highest correlation.^16^ Blood speckle tracking and echo-particle image velocimetry (echoPIV) are similar postprocessing strategies that can be applied to subsequent ultrasound frames to obtain velocity vectors. Both techniques divide the images into small blocks to estimate the mean displacement in each block based on chosen similarity index (usually cross-correlation or sum of squared differences).^17^^,^^18^ The main difference between blood speckle tracking and echoPIV is their signal source, where the former tracks the speckle patterns arising from red blood cell scattering, while echoPIV tracks intravenously injected microbubbles.^19^

From these five techniques, echoPIV seems ultimately suitable for imaging through a stent, since it uses ultrasound contrast agent (microbubbles) to enhance scattering of the moving fluid. The native blood scattering is very weak, resulting in low SNR. We expect that the stronger microbubble signal will overcome possible reduced signal intensity inside the stent.

EchoPIV has seen a considerable evolution since the first reported use of the technique in the medical field in 2004.^19^ In its early stage, several *in vitro* studies were performed to validate the technique,^20^^–^^23^ followed by *in vivo* studies in the left ventricle of the human heart.^22^^–^^24^ Due to the limited temporal resolution of the clinical ultrasound equipment, the frame rates were not sufficiently high to capture velocities greater than 0.4  m/s, which are present in ventricular flow. High frame rate (HFR) or ultrafast techniques overcome these problems.^25^ In 2015, the use of plane wave imaging to achieve HFRs for echoPIV was described.^26^ Since then, flow patterns in the carotid artery and the left ventricle were studied *in vitro*^27^^,^^28^ and *in vivo*.^25^^,^^29^^–^^32^ Moreover, HFR echoPIV was used in rabbits as proof of concept^26^ and in humans to study abdominal aortic flow patterns.^33^^,^^34^

To our knowledge, the performance of echoPIV in the presence of a stent has not yet been reported other than in our previous preliminary study.^35^ In this paper, we compare the performance of HFR plane wave echoPIV in a stented region with a nonstented region in a flow phantom. *In vitro* flow studies are beneficial because of a controlled environment to change specific parameters to characterize the performance of the system. Signal intensities are expected to be lower in the stent than outside the stent, since the ultrasound wavefront will interfere with the metal struts of the stent. Since the thickness of the stent struts (<100  μm) is smaller than the wavelength used this study (145 to 230  μm), no strong diffraction and interference patterns are expected inside the stent. Moreover, the openings of the stent mesh are larger (>300  μm) than the ultrasound wavelength, so the ultrasound wavefront will not be blocked by the stent. The expectation is that the stent will have no significant impact on the resolved flow inside this type of stent. This might be different in stents with thicker struts or a denser mesh. However, the effects of different stent types is out of the scope of this study. The effect of one type carotid stent on *in vitro* echoPIV performance will be verified in this study.

## 2 Materials and Methods

We evaluated the performance of echoPIV inside a stent in an *in vitro* flow setup. Several ultrasound acquisitions with variations from a single reference measurement were performed. Contrast ratios between stented and nonstented regions were obtained, and PIV analysis was performed.

### 2.1 Experimental Setup

**Fig. 1:**
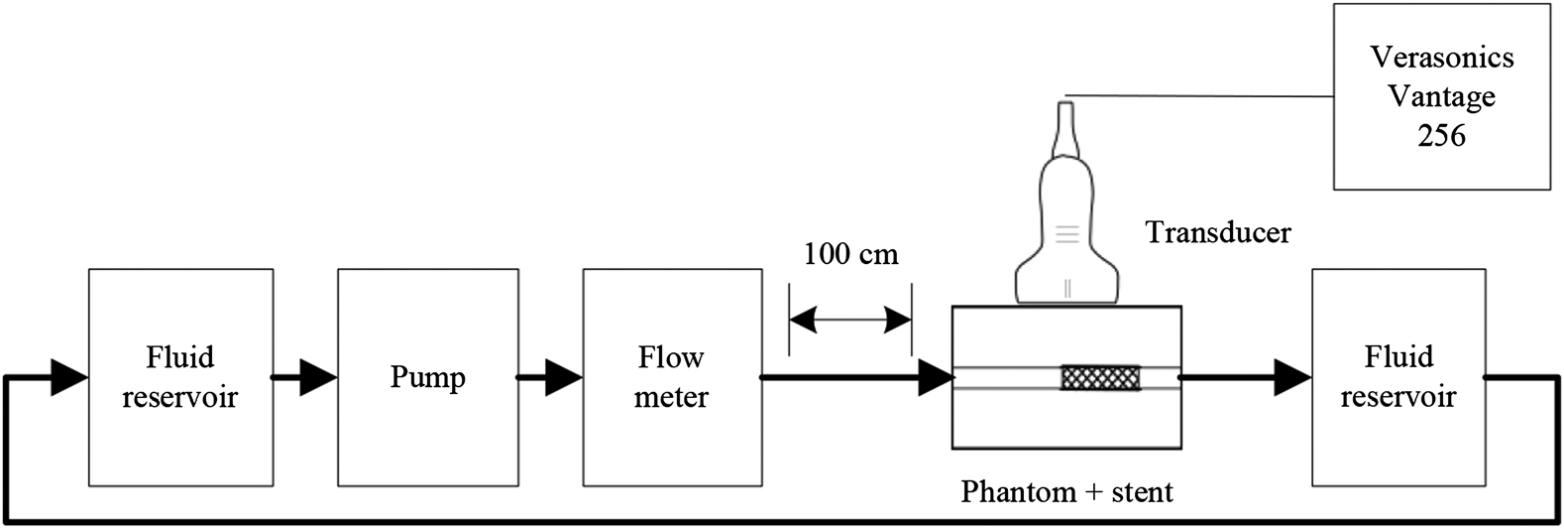
Schematic representation of the experimental flow setup.

Figure 1 shows a schematic representation of the experimental flow setup. A polyvinylalcohol-gel (PVA-gel) phantom was fabricated using 10 weight% PVA powder [Mw 85,000 to 124,000, 99+% hydrolyzed (363146), Sigma-Aldrich, St. Louis, Missouri] and 90 weight% commercial cooling agent (Coolant G12+/Longlife -30°C, AllRide). It was subjected to three freeze-thaw cycles of 17 h of freezing and 9 h of thawing. The phantom had a straight vessel design with a diameter of 8 mm and a length of 120 mm. Due to shrinking after fabrication, the diameter was 7.2 mm when measurements were performed. A 100-cm straight tube with an inner diameter of 8 mm was placed in front of the phantom to ensure fully developed flow in the phantom. A commercial self-expanding stent (Wallstent 40×10  mm, Boston Scientific, Marlborough, Massachusetts) was halved to fit in the phantom and one half was deployed halfway the vessel. In the stented part, the diameter of the vessel was enlarged to 7.8 mm due to outward pointing radial force of the stent.

The flow setup was filled with a blood mimicking fluid (BMF) consisting of water and 39 mass% glycerin. Theoretically, this mixture has a kinematic viscosity of $3.3\times 10^{-6}\;m^{2}/s$ at 20°C. The BMF was circulated through the setup using a gear pump with a continuous flow rate of 0.49  L/min on average, based on values of carotid artery flow rate reported in the literature^36^^–^^39^ and adapted for the diameter of the phantom. The flow rate was measured by an ultrasonic flowmeter (UF Ultrasonics Flow Meter, Cynergy3 Components, United Kingdom, 3% accuracy). The contrast agent, BR-14 microbubbles powder (Bracco Suisse SA, Geneva, Switzerland) was mixed by hand with 0.4-mL demineralized water, was added to 2 L BMF.

### 2.2 Ultrasound Measurement Protocol

Ultrasound acquisition was performed using a Verasonics Vantage 256 system (Verasonics Inc., Kirkland, Washington) with a Verasonics L12-3v linear array transducer. Two overlapping subapertures of 128 elements of the total of 192 elements were used for each angled plane wave acquisition (this was required because the probe was fitted with a single 128 channel interface to the Vantage system). In total, 250 frames were acquired per dataset. Using the reference settings (first row of Table 1), an uncompounded frame rate of 8789 frames per second was achieved (pulse repetition interval of individual frames was 113.78  μs). Automatic singular value decomposition (SVD) filtering was applied to suppress semistationary signals, while retaining the signal from the moving microbubbles.^33^ The angled frames were coherently compounded after SVD-filtering. No contrast-specific sequences such as pulse inversion or amplitude modulation were used.

**Table 1:** Measurement scheme showing all measurement settings in chronological order.

Measurement no.		Measurement description	No. of angles	Steering angle (deg)	Transmit frequency (MHz)	Stent position	Transmit voltage (V)
	R	Reference measurement	3	−10.5, 0, 10.5	9	Outflow	5
**1A**	**R1**	3 angles	3	—	—	—	—
**1B**		5 angles	5	—	—	—	—
**1C**		7 angles	7	—	—	—	—
**2A**		Opening angle 5.5	—	−5.5, 0, 5.5	—	—	—
**2B**	**R2**	Opening angle 10.5	—	−10.5, 0, 10.5	—	—	—
**2C**		Opening angle 15.5	—	−15.5, 0, 15.5	—	—	—
**3A**^a^		5 MHz transmit frequency	—	—	5	—	—
**3B**		7 MHz transmit frequency	—	—	7	—	—
**3C**	**R3**	9 MHz transmit frequency	—	—	9	—	—
**3D**		11 MHz transmit frequency	—	—	11	—	—
**4A**	**R4**	Outflow	—	—	—	Outflow	—
**4B**		Inflow	—	—	—	Inflow	—
**5A**	**R5**	5 V transmit	—	—	—	—	5
**5B**		10 V transmit	—	—	—	—	10
**5C**		15 V transmit	—	—	—	—	15

This table only shows the alterations relative to the reference settings as described in the first row. R1 to R5 all correspond to the reference settings.

a Measurement 3A is not included in PIV analysis (see Sec. 2.4).

Table 1 shows the settings of all acquisitions. We performed 15 measurements in 30 min, while BMF was continuously flowing without adding new contrast-agent. Within these 15 measurements, five subcategories can be distinguished. In each subcategory, one of the settings was changed, i.e., in the first category only the number of steering angles was altered. Each subcategory contained one measurement with settings equal to the so-called reference setting (Table 1, first row). We altered the number of steering angles, the angulation of the plane waves, the transmit frequency, and the transmit voltage. The ultrasound probe was positioned such that vessel was in the field of view with the stent located at the outflow side of the vessel (referred to as outflow in Table 1, column 6), except for measurement 4B, where we changed the position of the stent to the inflow side of the vessel (referred to as inflow in Table 1, column 6).

### 2.3 Analysis: Contrast Between Nonstented and Stented Regions

**Fig. 2:**
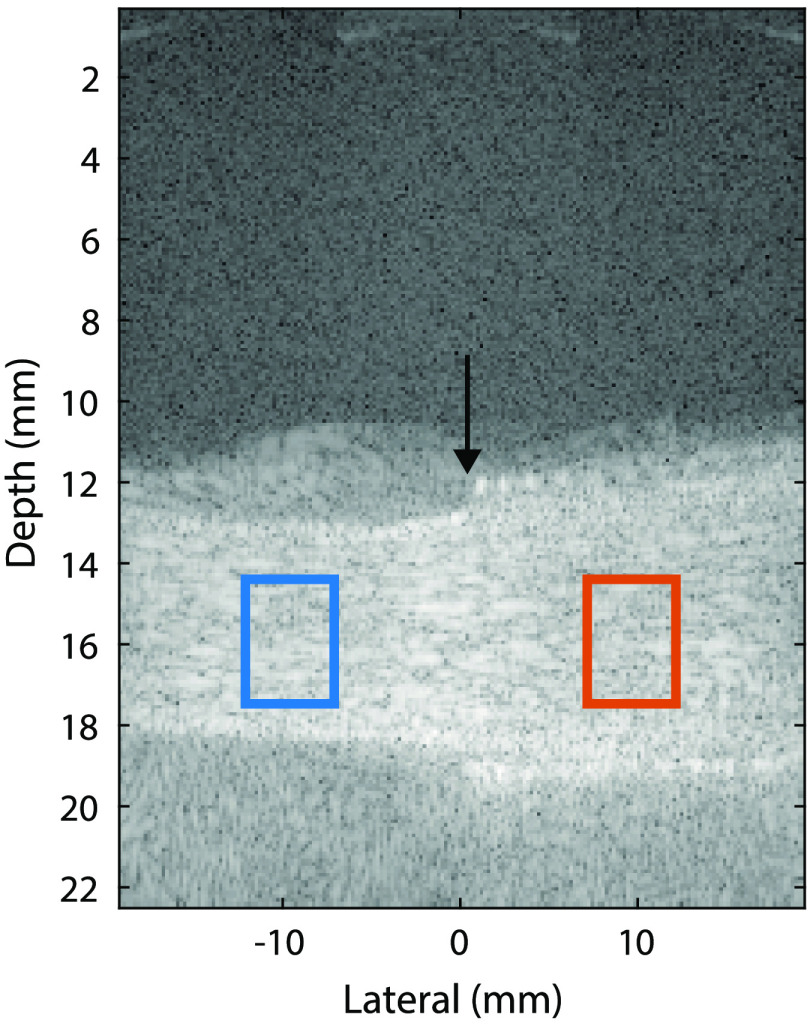
Time-averaged SVD-filtered B-mode frame of reference measurement 1 (R1) showing the ROIs of the nonstented region (blue) and stented region (orange) for contrast analysis. The stented area is from lateral position 0 mm to the right border of the image. The black arrow indicates the start of the stent.

The contrast ratio between the signal power inside and outside the stent was calculated. We manually selected a region of interest (ROI) both inside and outside the stent. Figure 2 shows that the ROIs are located at the same depth and with an equal distance from the center in the lateral direction. Although this figure shows the ROIs on the B-mode images, contrast is calculated using the IQ-data of the reconstructed, concatenated SVD-filtered frames. Contrast is calculated as

$$\text{Contrast}\;(\mathrm{dB})=10\;\log_{10}(\frac{P_{\mathrm{nostent}}}{P_{\text{stent}}}),$$ (1)

where the power outside the stented area is $P_{\text{nostent}}=I_{\text{nostent}}^{2}+Q_{\text{nostent}}^{2}$ and the power inside the stent is $P_{\text{stent}}=I_{\text{stent}}^{2}+Q_{\text{stent}}^{2}$. I and Q result from the IQ-data.

Contrast values were averaged over 250 frames.

### 2.4 Analysis: Particle Image Velocimetry

PIV analysis was performed in a modified version of PIVlab^40^ in MATLAB R2017b (The Mathworks Inc., Natick, Massachusetts) on the IQ-data of the compounded SVD-filtered frames. Two iterative interrogation areas of 32×32  pixels and 16×16  pixels with an overlap of 75% were applied on the first 100 frames of each measurement. Due to these interrogation areas, it is required to have pixels surrounding the ROI. In measurement 3A, the imaging depth was not sufficient to have the required number of pixels surrounding the contrast-enhanced vessel, therefore, PIV analysis could not be completed on measurement 3A (5 MHz transmit frequency). Outliers were removed and further postprocessing consisted of smoothing using Gaussian filters and moving averages.

Mean and peak velocity and correlation coefficients were computed and analyzed. The velocity data and correlation coefficients of each measurement were split into two separate regions: nonstented and stented (see Fig. 5). The vectors and correlation coefficients in-between the two regions were not included, to prevent ambiguity about which region the vectors belonged to.

### 2.5 Statistical Analysis: Contrast

Within each subcategory (1 to 5), mean contrast values were compared. Also, mean contrast values of the reference measurements were compared with each other.

A one-way ANOVA was conducted in SPSS (IBM Corp. Released 2019. IBM SPSS Statistics for Windows, Version 26.0. Armonk, New York: IBM Corp.) to compare contrast values between measurements with 3, 5, or 7 angles, with different steering angles, with 5, 7, 9, and 11 MHz transmit frequencies, with 5, 10, and 15 V transmit voltage and between the reference measurements. Tukey post hoc tests were conducted on these measurements to evaluate the individual differences. An independent samples T-test was conducted in SPSS to compare the measurements with the stent position on the inflow and on the outflow side.

### 2.6 Statistical Analysis: PIV

Statistical analysis on PIV results was performed on the five measurements that have equal settings, the reference measurements. PIV analysis resulted in estimated velocity vectors over 98 timeframes. For mean velocity calculation, all velocity magnitudes per region (stented and nonstented) were averaged over each frame. For maximum velocity, the maximum velocity magnitudes of each lateral position per region were averaged over each frame. This resulted in 98 mean and 98 maximum velocity magnitudes per region for each measurement.

Mean and maximum velocities outside the stent were compared with velocities in the stented region using independent-samples T-tests in SPSS. Mean and maximum velocities of each frame were taken as input. Correlation coefficients in and outside the stent were compared using the same approach.

Mean velocity inside the stent was compared with the theoretical mean velocity using one-sample T-test in SPSS. The axial theoretical velocity profile, derived from the Hagen–Poiseuille equation, can be described as

$$v(r)=v_{c}(1-\frac{r^{2}}{R^{2}}),$$ (2)

where v(r) is the velocity at radius r of the total vessel radius R, and $v_{c}$ is the center velocity. The equation for the theoretical mean velocity in the ultrasound image plane was derived by integrating Eq. (2) and dividing it by the diameter of the vessel:

$$v_{\text{mean}}=\frac{v_{c}}{2R}\int_{-R}^{+R}(1-\frac{r^{2}}{R^{2}})dr=\frac{2}{3}v_{c}.$$ (3)

We assumed $v_{c}$ to be the maximum velocity, which is calculated as

$$2\times (\frac{Q}{\pi R^{2}}),$$ (4)

where Q is the flow rate measured by the flow meter.

Using Eqs. (2)–(4), the theoretical mean and maximum velocity in the nonstented region (diameter is 7.2 mm) are 26.7 and 40.1  cm/s, respectively. The theoretical mean and maximum velocity in the stented region (diameter is 7.8 mm) are 22.8 and 34.2  cm/s, respectively.

## 3 Results

### 3.1 Contrast Between Nonstented and Stented Regions

**Fig. 3:**
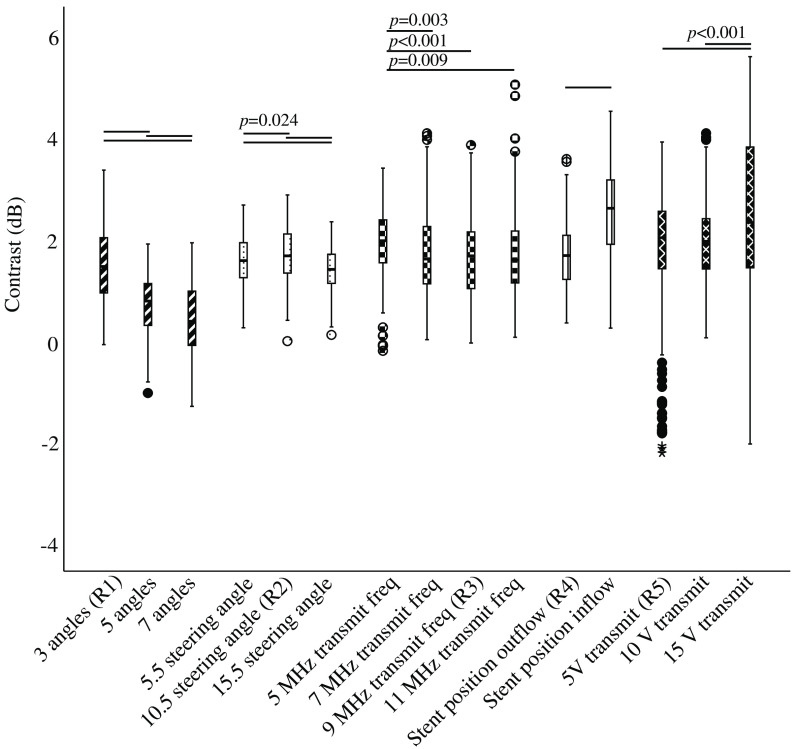
Boxplots of the contrast in all measurements. The different subcategories are clustered. Statistical differences within subcategories are indicated with lines above specific measurements. p≪0.001 applies for all comparisons, unless p-values are noted above the lines.

Mean contrast had a positive value in all measurements, so the intensity was higher in the nonstented region than in the stented part of the vessel (Fig. 3). Since mean contrast values were small (in the range of 0 to 3 dB), this difference was hardly visible in the time-averaged SVD frames (Fig. 2). The measurements using transmit voltage of 5 and 15 V showed a large spread compared with all other measurements.

Figure 3 shows boxplots of the contrast measurements where statistically significant differences within subgroups are indicated. One-way ANOVA showed a statistically significant difference (p<0.001) in contrast value within each subgroup. Increasing the number of angles resulted in statistically significant lower contrast, thus less difference between nonstented and stented areas. A steering angle of 15.5 deg shows lowest contrast value, compared with 5.5- and 10.5-deg steering angles. Transmit frequency had less influence on intensity between the two areas. The contrast was significantly lower when the stent was positioned on the outflow side compared with the inflow side. In the transmit voltage subgroup, the lowest contrast was seen using 5 or 10 V compared with 15 V.

**Fig. 4:**
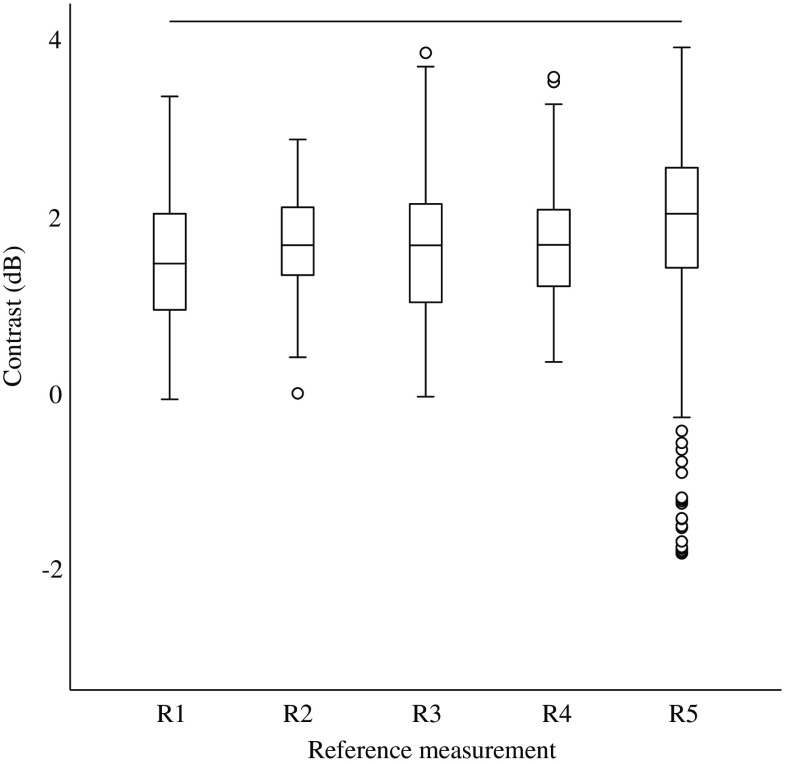
Boxplots of the contrast in the reference measurements. The line above R1 to R5 shows the statistically significant difference between these two measurements (p=0.006).

The reference measurements were also significantly different (p=0.012). Tukey’s post hoc test revealed that only reference measurements one and five differed significant (p=0.006) (Fig. 4).

### 3.2 Particle Image Velocimetry

**Fig. 5:**
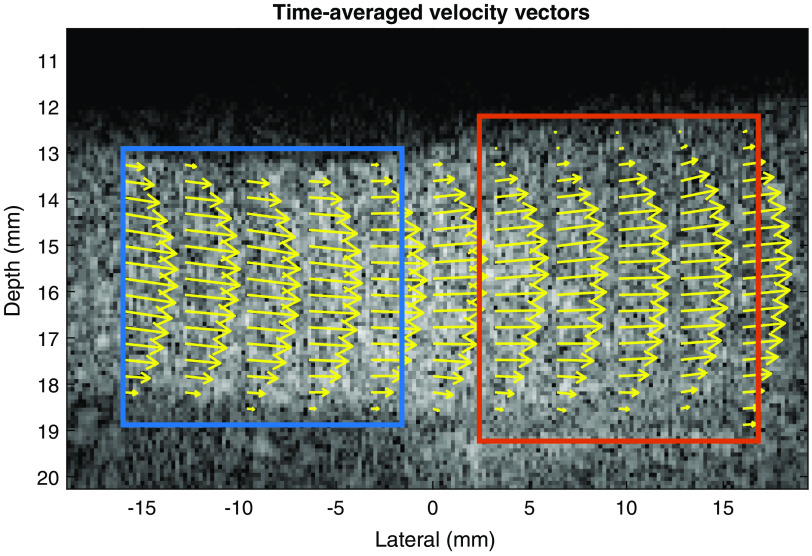
Time-averaged velocity vectors (yellow arrows) of reference measurement 1 in nonstented area (blue) and stented area (orange). The velocity vectors are projected on a single SVD filtered frame.

PIV analysis showed laminar flow patterns in the entire vessel (Fig. 5). Notice the increased diameter in the stented region. Mean and maximum velocities were lower in the stented region than in the nonstented region (Table 2). The difference between the two regions was larger for the mean velocity (4.32  cm/s) than for the maximum velocity (2.58  cm/s). Correlation coefficients were consistently lower in stented region (0.72) than in nonstented region (0.82) (Table 2).

**Table 2:** Velocities of each measurement in cm/s for nonstented and stented regions.

Measurement	(Reference measurement)	Nonstented region			Stented region		
		Mean velocity cm/s (SD)	Maximum velocity cm/s (SD)	Correlation coefficient (SD)	Mean velocity cm/s (SD)	Maximum velocity cm/s (SD)	Correlation coefficient (SD)
**1A**	**(R1)**	26.51 (0.28)	36.51 (0.32)	0.80 (0.01)	21.88 (0.24)	33.98 (0.38)	0.71 (0.01)
**1B**		26.47 (0.15)	36.89 (0.32)	0.84 (0.01)	21.99 (0.19)	34.55 (0.19)	0.77 (0.01)
**1C**		26.94 (0.11)	37.86 (0.29)	0.82 (0.01)	22.54 (0.12)	35.63 (0.25)	0.75 (0.01)
**2A**		26.93 (0.19)	37.97 (0.33)	0.87 (0.01)	22.18 (0.20)	35.41 (0.48)	0.78 (0.01)
**2B**	**(R2)**	27.21 (0.18)	36.29 (0.79)	0.76 (0.01)	22.41 (0.44)	33.72 (0.51)	0.67 (0.01)
**2C**		26.52 (0.40)	34.84 (0.54)	0.76 (0.01)	21.84 (0.33)	32.25 (0.47)	0.67 (0.02)
**3B**		26.38 (0.22)	37.17 (0.40)	0.84 (0.01)	21.88 (0.20)	34.51 (0.32)	0.76 (0.02)
**3C**	**(R3)**	26.25 (0.21)	37.21 (0.54)	0.82 (0.01)	22.64 (0.19)	34.93 (0.42)	0.73 (0.02)
**3D**		26.29 (0.40)	37.60 (0.65)	0.78 (0.03)	22.31 (0.47)	34.81 (0.69)	0.67 (0.03)
**4A**	**(R4)**	27.05 (0.20)	37.80 (0.73)	0.82 (0.01)	22.70 (0.25)	35.26 (0.73)	0.71 (0.02)
**4B**		24.52 (0.34)	34.12 (0.70)	0.85 (0.01)	20.79 (0.21)	31.40 (0.32)	0.72 (0.02)
**5A**	**(R5)**	27.24 (0.22)	37.25 (0.29)	0.82 (0.01)	22.76 (0.27)	34.20 (0.36)	0.70 (0.01)
**5B**		26.66 (0.25)	37.00 (0.64)	0.83 (0.01)	22.59 (0.22)	33.75 (0.46)	0.72 (0.02)
**5C**		26.07 (0.17)	35.13 (0.75)	0.82 (0.03)	21.95 (0.22)	33.08 (0.82)	0.72 (0.03)
**Average**		26.50 (0.70)	36.69 (1.28)	0.82 (0.03)	22.18 (0.57)	34.11 (1.26)	0.72 (0.04)
**Average R1-R5**		26.85 (0.45)	37.01 (0.79)	0.80 (0.02)	22.48 (0.43)	34.42 (0.77)	0.71 (0.02)
**Theoretical**		26.7	40.1	—	22.8	34.2	—

SD, standard deviation.

PIV-derived velocities were compared with theoretical values. The mean velocity inside the stent (22.48  cm/s, SD=0.435) was significantly different from the theoretical mean velocity (22.8  cm/s); p≪0.001.

**Fig. 6:**
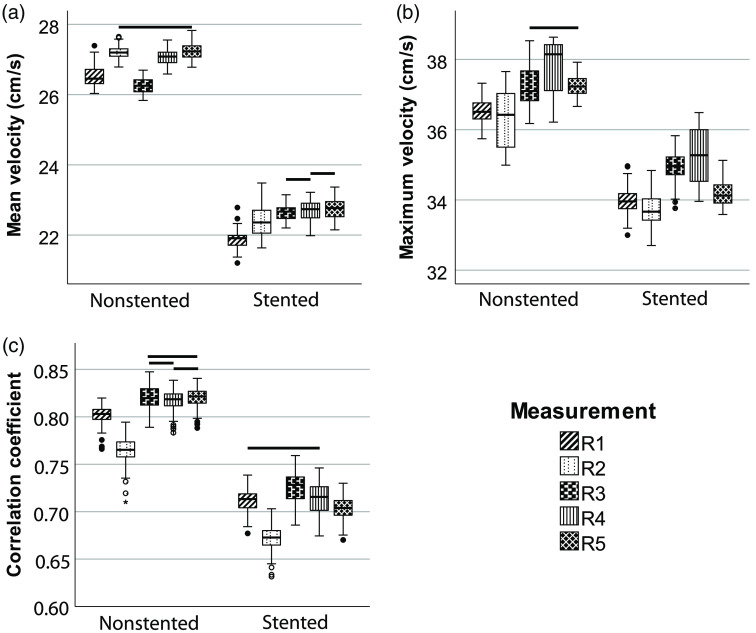
Boxplots of reference measurements showing (a) mean velocities, (b) maximum velocities, and (c) correlation coefficients for nonstented and stented regions. The horizontal lines above the boxplots indicate no significant difference (p>0.05).

Comparison of the five reference measurements resulted in significant differences in mean and maximum velocities and in correlation coefficients. Some measurements were not statistically significantly different as depicted the boxplots of Fig. 6.

## 4 Discussion

In this study, we have evaluated the effect of a carotid artery stent on contrast agent signal intensity and the accuracy of echoPIV-derived velocities inside the stented region. To evaluate the difference in signal intensity between the stented and nonstented regions, the contrast between these two regions was calculated. The contrast analysis shows that, on average, signal intensity is lower in the stented region than outside the stent. PIV analysis resulted in lower velocity values and correlation coefficients in the stented region than in the nonstented region. To analyze the difference from a ground truth, the velocity in the stent was compared with an estimated theoretical value. A statistically significant difference was seen between mean velocity in the stent and the theoretical mean velocity.

This study is a continuation of our previous preliminary experiments on the effect of a stent on HFR echoPIV.^35^ To the best of our knowledge, these are the first studies that report about HFR echoPIV in a stent. EchoPIV is an emerging technique to study blood flow patterns in detail, and it might be ultimately suitable for analysis of *in vivo* stent performance, because the enhanced scattering of the moving fluid might overcome a possible reduction of image quality caused by the stent. The setup has been improved since the previous study. We now used a linear array transducer with a shorter elevation depth and a better ultrasound-eligible phantom material, to overcome the limitations reported in that study. For this study, we have added the analysis of several settings on the effect of imaging through a stent and elaborated on contrast and velocity estimations. The decreased correlation coefficients and signal intensities in the stent are consistent with data obtained in our previous study.^35^

We conclude that contrast measurements are reproducible over time, however, the results of velocity analysis seem not that reproducible. This is based on the five measurements with equal settings, the so-called reference measurements. Contrast was only significantly different between reference measurements 1 and 5 (Fig. 4), while the results of PIV analysis showed significant differences between most reference measurements (Fig. 6). A possible explanation for this lack of consistency is the variability in flow control, as the pump operated within a range of 0.46 and 0.52  L/min. Moreover, contrast is a ratio of two intensity values, while velocity and correlation coefficient are absolute values, which are more prone to subtle changes.

From literature and previous experiments, we know that microbubbles are disrupted by the pressure of the ultrasound beam.^33^^,^^41^^,^^42^ Theoretically, the microbubble signal would be lower at the outflow side of the vessel, because the microbubbles are exposed longer to the ultrasound beam. In our reference setup, the stent is positioned at the outflow side of the vessel, therefore we cannot distinguish if a lower signal strength in the stented part would be caused by the stent or by disrupted bubbles. Measurement 4 is performed to check this. If bubbles were measurably destroyed during one measurement, we would expect to see a lower value for contrast in measurement 4B (stent on the inflow side). In that case, the signal intensity in the stent would be higher (more bubbles), and the signal intensity outside the stent would be lower (less bubbles). However, our results show a significantly increased contrast value in measurement 4B. Therefore, we do not assume that the difference in contrast between the two regions in all other measurements are due to destroyed bubbles at the outflow side. The decreased correlation coefficients in the stented part, also present in measurement 4B, confirm these thoughts.

There are differences between stented and nonstented regions in both contrast measurements and PIV outcomes. On average, the contrast has a positive value, which means that the signal intensity is lower in the stent than outside the stent. This is a logical result of imaging through a stent, because imaging through a grid of metal wire can attenuate the ultrasound waves, resulting in less enhanced signal in the stented region. Mean and maximum velocities are also lower in the stented region of the phantom. Since the diameter of the phantom was increased by the forces of the stent, we expected to see lower velocities in the stent. Due to this diameter difference, it is difficult to determine if the metal wires of the stent also contributed to the decrease in velocity estimation. Correlation coefficients were also significantly lower in the stented region. As correlation coefficients indicate quality of PIV analysis, we can conclude that quality of velocity estimation decreases when imaging through a stent. However, the quality of PIV in a stented vessel is acceptable, since PIV analysis still showed laminar flow profiles and plausible velocity values.

PIV settings, such as the size of the interrogation area, should be carefully chosen. These settings depend on, for example, expected velocities, pixel size, and point spread function. We used two iterative interrogation windows of 32×32  pixels and 16×16  pixels, commonly used sizes and 2D Gaussian peak fitting as subpixel displacement estimation method. The maximum velocity of ∼40  cm/s and the effective frame rate of ∼3  kHz results in a displacement of ∼0.7  pixel per frame. This satisfies the design rule of a displacement less than a quarter of the interrogation area.^43^ However, the downside of subpixel displacement is that the results are more prone to error. Possible solutions to improving accuracy would be skipping frames for PIV analysis (increasing the effective frame-to-frame displacement to over 1 pixel) or increasing the resolution of the beamforming grid (decreasing pixel size—however, this would incur higher computation costs).

Comparison with theoretical velocities and profiles is difficult in this set of measurements. Mean velocity in the stented region differed significantly from the theoretical value. It turns out that comparison with a theoretical profile is complicated, because the field of view is probably not in the center of the vessel. This is based on the observation that the diameter in the images does not correspond to the real diameter. Moreover, the slice thickness, and thereby averaging of velocities, contributes to the differences with theoretical velocities. Due to these differences, we cannot define a certain offset for velocities obtained by echoPIV imaging through a stent. However, there is also no definition of such a deviation or correction factor for clinical Duplex in carotid stents. Some studies showed increased peak systolic velocities in the stent,^44^^–^^46^ whereas others showed no difference^47^ or stent-dependent deviations.^48^

**Fig. 7:**
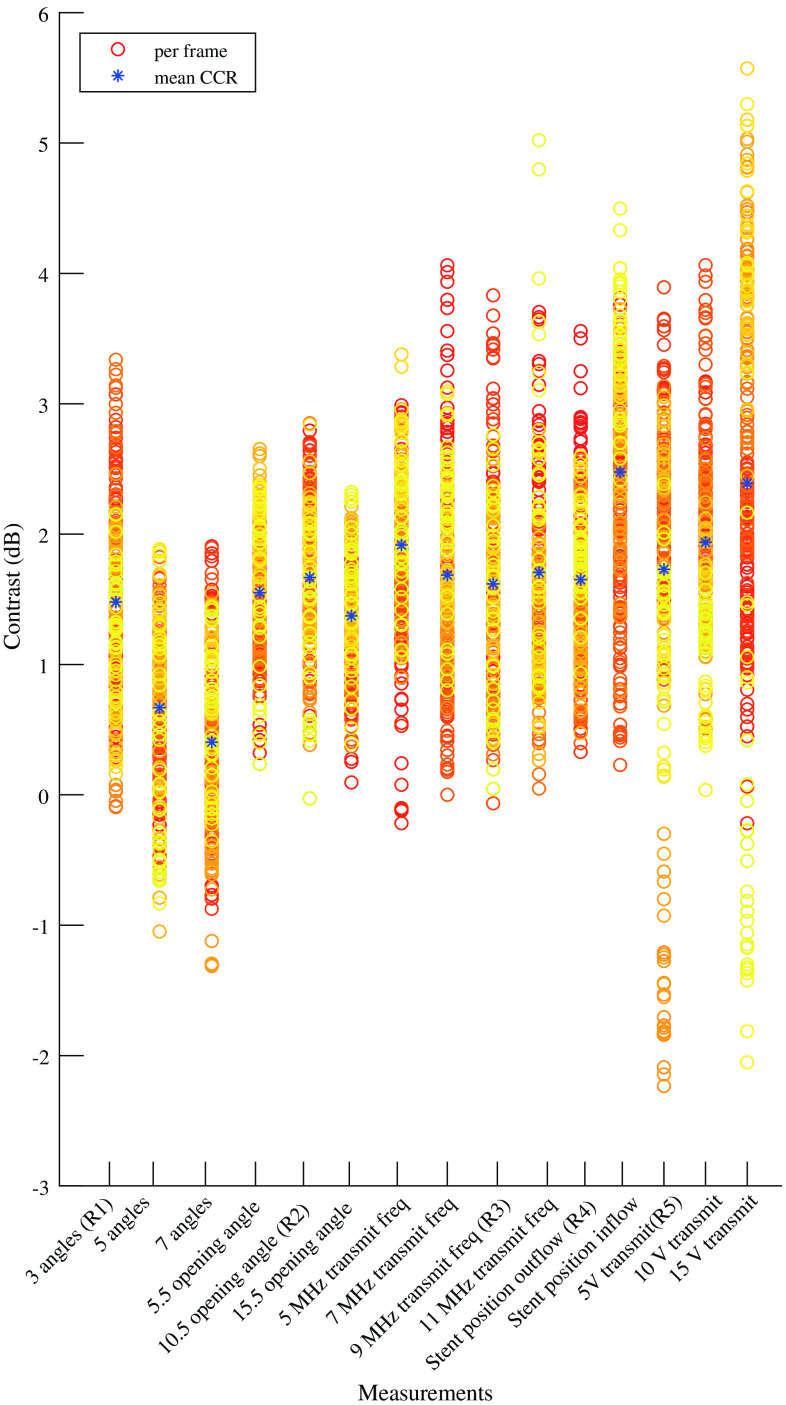
Scatterplot of contrast values in dB per measurement. Each circle represents a contrast measurement of one frame. The color scale ranges from red [timepoint 1 (= 0 s)] to yellow [latest timepoint (=0.17 s)].

A large variance in contrast is visible in measurements 5A and 5C of the transmit voltage subcategory, with standard deviations of 1.25 and 1.59 dB, respectively (Fig. 3). In comparison, the pooled standard deviation of all contrast measurements is 0.97 dB. The larger spread in these two measurements could be due to alterations to the setup because of changing the position of the stent in measurement 4B. However, measurement 5B has a standard deviation of 0.74 dB, which is significantly lower and does not support this explanation for large spread. Another explanation might be bubble disruption, as we know this occurs at higher transmit voltages.^33^^,^^41^^,^^42^ The increased contrast in the measurement of 15 V transmit voltage can be explained by the severe bubble disruption, as we expect to see lower signal intensity at the outflow side of the vessel after bubble disruption at high pressure. Also, during the measurement, bubble concentration will become lower, this can explain the larger spread in the measurement. We see an increase of contrast value over time for measurement 5C; however, this trend is not present in other measurements (Appendix, Fig. 7). Therefore, we cannot draw conclusions on the role of bubble disruption in the larger spread at higher transmit voltages.

Our measurements show a decrease in contrast values when the number of angles increases (measurements 1A-C, Fig. 3). If the stent is seen as an obstruction to the ultrasound signal, then approaching the vessel from different angles would result in less signal loss. The more angles are used, the more signal will reach the bubbles, and therefore lower contrast values will be seen.

A limitation of this study is the restriction to one stent type. Future *in vitro* studies should focus on the effects of HFR echoPIV in other stent types, since the cell areas (open structures of the stents) differ between different stent types. This may influence the ultrasound signal protrusion through the stent. Another limitation is the restricted comparison with the theoretical flow profiles. As indicated before, the possible cause of the deviation from the theoretical flow profile, especially in the nonstented part, is the positioning of the ultrasound transducer. This will remain a critical and challenging issue in future experiments. However, since the diameter of the vessel was enlarged by the stent and the transition zone was short, we do not know for sure if there was a fully developed flow profile in the stent. The stent itself, creating a “rough surface,” will also influence the flow profile. To investigate the effect of the metal of the stent on the ultrasound performance, the stent should ideally not change the diameter nor the surface roughness of the vessel. For comparison with *in vivo* situations, pulsatile flow conditions and comparison with theoretical Womersley profiles are highly recommended. Besides the pulsatile flow, *in vivo* situations are far more complex than this simplified ideal and straight vessel without bifurcation, which may also be of influence of the performance of echoPIV in *in vivo* stents.

## 5 Conclusions

This study shows that velocity profiles in the presence of a stent can be measured using echoPIV in an *in vitro* setup. The stent causes a decrease in signal intensities of contrast agent. This results in decreased correlation coefficients after PIV analysis. Both observations correspond to our expectations that a Wallstent causes the image quality to decrease. However, our results show that the quality with a correlation coefficient of 0.7 is still sufficient to perform PIV analysis. Velocity vectors of continuous flow within clinical range of carotid flow rates (0.49  L/min) can be obtained in a stented phantom. Future work should focus on pulsatile flow conditions and *in vivo* effects of stent on the quality of echoPIV.

## 6 Appendix A: Contrast Values Over Time

We visualized contrast for each frame separately, to check whether contrast increases because of bubble disruption. Bubble disruption occurs at higher transmit voltages. Therefore, measurement 5 is of interest to see this effect. Figure 7 shows an increase in contrast over time in measurement 5C (transmit voltage 15 V). However, a decrease over time is visible in measurement 5B (transmit voltage 10 V).

## References

[1] Brott T. G. et al., “Stenting versus endarterectomy for treatment of carotid-artery stenosis,” N. Engl. J. Med. 363(1), 11–23 (2010). NEJMAG 0028-4793 10.1056/NEJMoa0912321 20505173 PMC2932446

[2] Bonati H. et al., “Percutaneous transluminal balloon angioplasty and stenting for carotid artery stenosis,” Cochrane database Syst. Rev. 9(9), CD000515 (2012). 10.1002/14651858.CD000515 22972047

[3] De Borst G. J. Naylor A. R., “In the end, it all comes down to the beginning!” Eur. J. Vasc. Endovasc. Surg. 50(3), 271–272 (2015). 10.1016/j.ejvs.2015.04.013 25957820

[4] Bonati L. H. et al., “Long-term outcomes after stenting versus endarterectomy for treatment of symptomatic carotid stenosis: the International Carotid Stenting Study (ICSS) randomised trial,” Lancet 385(9967), 529–538 (2015). LANCAO 0140-6736 10.1016/S0140-6736(14)61184-3 25453443 PMC4322188

[5] Wentzel J. J. et al., “Geometry guided data averaging enables the interpretation of shear stress related plaque development in human coronary arteries,” J. Biomech. 38(7), 1551–1555 (2005). JBMCB5 0021-9290 10.1016/j.jbiomech.2004.06.022 15922767

[6] Klemm T. et al., “MR imaging in the presence of vascular stents: a systematic assessment of artifacts for various stent orientations, sequence types, and field strengths,” J. Magn. Reson. Imaging 12(4), 606–615 (2000). 10.1002/1522-2586(200010)12:4<606::AID-JMRI14>3.0.CO;2-J 11042644

[7] Park M. Y. et al., “Effect of beam-flow angle on velocity measurements in modern Doppler ultrasound systems,” Am. J. Roentgenol. 198(5), 1139–1143 (2012). AJROAM 0092-5381 10.2214/AJR.11.7475 22528905

[8] Acker J. D. et al., “Duplex carotid ultrasound,” Neuroradiology 28, 608–617 (1986). 10.1007/BF00344109 2948133

[9] Fox M. D., “Multiple crossed-beam ultrasound Doppler,” IEEE Trans. Sonics Ultrason. 25(5), 281–286 (1978). IESUAU 0018-9537 10.1109/T-SU.1978.31028

[r10] Dunmire B. et al., “Cross-beam vector Doppler ultrasound for angle-independent velocity measurements,” Ultrasound Med. Biol. 26(8). 1213–1235 (2000). USMBA3 0301-5629 10.1016/S0301-5629(00)00287-8 11120358

[r11] Ricci S. Bassi L. Tortoli P., “Real-time vector velocity assessment through multigate Doppler and plane waves,” IEEE Trans. Ultrason. Ferroelectr. Freq. Control 61(2), 314–324 (2014). ITUCER 0885-3010 10.1109/TUFFC.2014.6722616 24474137

[12] Ricci S. et al., “Real-time blood velocity vector measurement over a 2-D region,” IEEE Trans. Ultrason. Ferroelectr. Freq. Control 65(2), 201–209 (2018). ITUCER 0885-3010 10.1109/TUFFC.2017.2781715 29389652

[13] Jensen J. A. Munk P., “A new method for estimation of velocity vectors,” IEEE Trans. Ultrason. Ferroelectr. Freq. Control 45(3), 837–851 (1998). ITUCER 0885-3010 10.1109/58.677749 18244236

[r14] Salles S. et al., “2-D arterial wall motion imaging using ultrafast ultrasound and transverse oscillations,” IEEE Trans. Ultrason. Ferroelectr. Freq. Control 62(6), 1047–1058 (2015). ITUCER 0885-3010 10.1109/TUFFC.2014.006910 26067039

[15] Lenge M. et al., “Plane-wave transverse oscillation for high-frame-rate 2-D vector flow imaging,” IEEE Trans. Ultrason. Ferroelectr. Freq. Control 62(12), 2126–2137 (2015). ITUCER 0885-3010 10.1109/TUFFC.2015.007320 26670852

[16] Kortbek J. Jensen J. A., “Determination of velocity vector angles using the directional cross-correlation method,” IEEE Trans. Ultrason. Ferroelectr. Freq. Control 53(11), 2036–2049 (2006). ITUCER 0885-3010 10.1109/TUFFC.2006.144 17091840

[17] Trahey G. E. Allison J. W. Von Ramm O. T., “Angle independent ultrasonic detection of blood flow,” IEEE Trans. Biomed. Eng. BME-34(12), 965–967 (1987). IEBEAX 0018-9294 10.1109/TBME.1987.325938 2961682

[18] Bohs L. N. et al., “Speckle tracking for multi-dimensional flow estimation,” Ultrasonics 38(1), 369–375 (2000). ULTRA3 0041-624X 10.1016/S0041-624X(99)00182-1 10829690

[19] Kim H. B. Hertzberg J. R. Shandas R., “Development and validation of echo PIV,” Exp. Fluids 36(3), 455–462 (2004). EXFLDU 0723-4864 10.1007/s00348-003-0743-5

[20] Liu L. et al., “Development of a custom-designed echo particle image velocimetry system for multi-component hemodynamic measurements: system characterization and initial experimental results,” Phys. Med. Biol. 53(5), 1397–1412 (2008). PHMBA7 0031-9155 10.1088/0031-9155/53/5/015 18296769

[r21] Westerdale J. et al., “Flow velocity vector fields by ultrasound particle imaging velocimetry,” J. Ultrasound Med. 30(2), 187–195 (2011). JUMEDA 0278-4297 10.7863/jum.2011.30.2.187 21266556

[r22] Prinz C. et al., “Can echocardiographic particle image velocimetry correctly detect motion patterns as they occur in blood inside heart chambers? A validation study using moving phantoms,” Cardiovasc. Ultrasound 10(1), 1–10 (2012). 10.1186/1476-7120-10-24 22672727 PMC3439370

[r23] Kheradvar A. et al., “Echocardiographic particle image velocimetry: a novel technique for quantification of left ventricular blood vorticity pattern,” J. Am. Soc. Echocardiogr. 23(1), 86–94 (2010). JSECEJ 0894-7317 10.1016/j.echo.2009.09.007 19836203

[24] Abe H. et al., “Contrast echocardiography for assessing left ventricular vortex strength in heart failure: a prospective cohort study,” Eur. Heart J. Cardiovasc. Imaging 14(11), 1049–1060 (2013). 10.1093/ehjci/jet049 23588788

[25] Voorneveld J. et al., “High-frame-rate echo-particle image velocimetry can measure the high-velocity diastolic flow patterns,” Circ. Cardiovasc. Imaging 12(4), e008856 (2019). 10.1161/CIRCIMAGING.119.008856 30939921

[26] Leow C. H. et al., “Flow velocity mapping using contrast enhanced high-frame-rate plane wave ultrasound and image tracking: methods and initial *in vitro* and *in vivo* evaluation,” Ultrasound Med. Biol. 41(11), 2913–2925 (2015). USMBA3 0301-5629 10.1016/j.ultrasmedbio.2015.06.012 26275971

[27] Leow C. H. Tang M. X., “Spatio-temporal flow and wall shear stress mapping based on incoherent ensemble-correlation of ultrafast contrast enhanced ultrasound images,” Ultrasound Med. Biol. 44(1), 134–152 (2018). USMBA3 0301-5629 10.1016/j.ultrasmedbio.2017.08.930 29037843

[28] Voorneveld J. et al., “High frame rate ultrasound particle image velocimetry for estimating high velocity flow patterns in the left ventricle,” IEEE Trans. Ultrason. Ferroelectr. Freq. Control 65(12), 2222–2232 (2017). ITUCER 0885-3010 10.1109/TUFFC.2017.2786340 29990263

[29] Nie L. et al., “High-frame-rate contrast-enhanced echocardiography using diverging waves: 2-D motion estimation and compensation,” IEEE Trans. Ultrason. Ferroelectr. Freq. Control 66(2), 359–371 (2019). ITUCER 0885-3010 10.1109/TUFFC.2018.2887224 30575531

[r30] Toulemonde M. E. G. et al., “High frame-rate contrast echocardiography: in-human demonstration,” JACC Cardiovasc. Imaging 11(6), 923–924 (2018). 10.1016/j.jcmg.2017.09.011 29248652

[r31] Gates P. E. et al., “Measurement of wall shear stress exerted by blowing blood in the human carotid artery: ultrasound Doppler velocimetry and echo particle image velocimetry,” Ultrasound Med. Biol. 44(7), 1392–1401 (2018). USMBA3 0301-5629 10.1016/j.ultrasmedbio.2018.02.013 29678322 PMC5960638

[32] Gurung A. et al., “Echo particle image velocimetry for estimation of carotid artery wall shear stress: repeatability, reproducibility and comparison with phase-contrast magnetic resonance imaging,” Ultrasound Med. Biol. 43(8), 1618–1627 (2017). USMBA3 0301-5629 10.1016/j.ultrasmedbio.2017.03.020 28501327

[33] Voorneveld J. et al., “High-frame-rate contrast-enhanced ultrasound for velocimetry in the human abdominal aorta,” IEEE Trans. Ultrason. Ferroelectr. Freq. Control 65(12), 2245–2254 (2018). ITUCER 0885-3010 10.1109/TUFFC.2018.2846416 29994206

[34] Engelhard S. et al., “High-frame-rate contrast-enhanced US particle image velocimetry in the abdominal aorta: first human results,” Radiology 289(1), 119–125 (2018). RADLAX 0033-8419 10.1148/radiol.2018172979 30015586

[35] Hoving A. M. et al., “*In vitro* high-frame-rate contrast-enhanced ultrasound particle image velocimetry in a carotid artery stent,” Proc. SPIE 10580, 105800A (2018). PSISDG 0277-786X 10.1117/12.2293669

[36] Cibis M. et al., “Wall shear stress calculations based on 3D cine phase contrast MRI and computational fluid dynamics: a comparison study in healthy carotid arteries,” NMR Biomed. 27(7), 826–834 (2014). 10.1002/nbm.3126 24817676

[r37] Schoning M. Walter J. Scheel P., “Estimation of cerebral blood flow through color duplex sonography of the carotid and vertebral arteries in healthy adults,” Stroke 25(1), 17–22 (1994). SJCCA7 0039-2499 10.1161/01.STR.25.1.17 8266366

[r38] Lee S. W. et al., “Geometry of the carotid bifurcation predicts its exposure to disturbed flow,” Stroke 39(8), 2341–2347 (2008). SJCCA7 0039-2499 10.1161/STROKEAHA.107.510644 18556585

[39] Hoi Y. et al., “Characterization of volumetric flow rate waveforms at the carotid bifurcations of older adults,” Physiol. Meas. 31(3), 291–302 (2010). PMEAE3 0967-3334 10.1088/0967-3334/31/3/002 20086276 PMC2943236

[40] Thielicke W. Stamhuis E. J., “PIVlab – towards user-friendly, affordable and accurate digital particle image velocimetry in MATLAB,” J. Open Res. Software 2, e30 (2014). 10.5334/jors.bl

[41] Toulemonde M. Eckersley R. J. Tang M. X., “High frame rate contrast enhanced echocardiography: microbubbles stability and contrast evaluation,” in IEEE Int. Ultrason. Symp. (2017). 10.1109/ULTSYM.2017.8092082

[42] Couture O. Fink M. Tanter M., “Ultrasound contrast plane wave imaging,” IEEE Trans. Ultrason. Ferroelectr. Freq. Control 59(12), 2676–2683 (2012). ITUCER 0885-3010 10.1109/TUFFC.2012.2508 23221216

[43] Keane R. D. Adrian R. J., “Optimization of particle image velocimeters. Part I: Double pulsed systems,” Meas. Sci. Technol. 1, 1202–1215 (1990). PSISDG 0277-786X 10.1088/0957-0233/1/11/013

[44] Lal B. K. et al., “Duplex ultrasound velocity criteria for the stented carotid artery,” J. Vasc. Surg. 47(1), 63–73 (2008). 10.1016/j.jvs.2007.09.038 18178455

[r45] Stanziale S. F. et al., “Determining in-stent stenosis of carotid arteries by duplex ultrasound criteria,” J. Endovasc. Ther. 12(3), 346–353 (2005). 10.1583/04-1527.1 15943510

[46] Naylor A. R. et al., “Management of atherosclerotic carotid and vertebral artery disease: 2017 clinical practice guidelines of the ESVS,” Eur. J. Vasc. Endovasc. Surg. 55(1), 3–81 (2018). 10.1016/j.ejvs.2017.06.021 28851594

[47] Bosch F. T. M. et al., “Optimal cut-off criteria for duplex ultrasound compared with computed tomography angiography for the diagnosis of restenosis in stented carotid arteries in the international carotid stenting study,” Eur. Stroke J. 2(1), 37–45 (2017). 10.1177/2396987316678361 31008301 PMC6453175

[48] Spies C. et al., “Carotid artery stent type influences duplex ultrasonography derived peak systolic velocity: findings of an *in-vitro* model,” Catheter. Cardiovasc. Interv. 70(2), 309–315 (2007). 10.1002/ccd.21224 17630677

